# Nocturnal pulse oximetry for the detection and prediction of acute mountain sickness: An observational study

**DOI:** 10.1113/EP091691

**Published:** 2024-09-15

**Authors:** Kelsey E. Joyce, Kimberly Ashdown, John P. Delamere, Chris Bradley, Christopher T. Lewis, Abigail Letchford, Rebekah A. I. Lucas, Will Malein, Owen Thomas, Arthur R. Bradwell, Samuel J. E. Lucas

**Affiliations:** ^1^ School of Sport, Exercise and Rehabilitation Sciences University of Birmingham Birmingham UK; ^2^ Birmingham Medical Research Expeditionary Society University of Birmingham Birmingham UK; ^3^ Occupational Performance Research Group University of Chichester Chichester UK; ^4^ Medical School University of Birmingham Birmingham UK; ^5^ Department of Anaesthesia Ysbyty Gwynedd Bangor UK; ^6^ Greysleydale Healthcare Centre Swadlincote UK; ^7^ Department of Anaesthesia Ninewells Hospital Dundee UK; ^8^ Department of Anaesthesia Royal Gwent Hospital, NHS Direct Wales Newport UK

**Keywords:** high altitude, hypoxia, peripheral oxygenation, sleep

## Abstract

Acute mountain sickness (AMS) is a well‐studied illness defined by clinical features (e.g., headache and nausea), as assessed by the Lake Louise score (LLS). Although obvious in its severe form, early stages of AMS are poorly defined and easily confused with common travel‐related conditions. Measurement of hypoxaemia, the cause of AMS, should be helpful, yet to date its utility for identifying AMS susceptibility remains unclear. This study quantified altitude‐induced hypoxaemia in individuals during an ascent to 4800 m to determine the utility of nocturnal pulse oximetry measurements for prediction of AMS. Eighteen individuals (36 ± 16 years of age) ascended to 4800 m over 12 days. Symptomology of AMS was assessed each morning via LLS criteria, with participants categorized as either AMS‐positive (LLS ≥ 3 with headache) or AMS‐negative. Overnight peripheral oxygen saturations (ov‐$S_{pO_{2}}$) were recorded continuously (1 Hz) using portable oximeters. Derivatives of these recordings were compared between AMS‐positive and ‐negative subjects (Mann–Whitney *U*‐test). Exploratory analyses (Pearson's) were conducted to investigate relationships between overnight parameters and AMS severity. Overnight derivatives, including ov‐$S_{pO_{2}}$, heart rate/ov‐$S_{pO_{2}}$, variance, oxygen desaturation index, hypoxic burden and total sleep time at <80% $S_{pO_{2}}$, all differed significantly between AMS‐positive and ‐negative subjects (all *P *< 0.01), with cumulative/relative frequency plots highlighting these differences visually. Exploratory analysis revealed that ov‐$S_{pO_{2}}$ from 3850 m was correlated with peak LLS at 4800 m (*r* = 0.58–0.61). The findings highlight the potential for overnight oximetry to predict AMS susceptibility during ascent to high altitude. Further investigation is required to develop, evaluate and optimize predictive models to improve AMS management and prevention.

## Abstract (highlights)

- **What is the central question of this study?**
  Early stages of acute mountain sickness are poorly defined, with existing assessment methods for the illness being highly ineffective. Can overnight pulse oximetry be used as a detection and prediction tool for deterioration in acute mountain sickness?
- **What is the main finding and its importance?**
  Overnight oximetry measurements from 3850 m were significantly correlated with acute mountain sickness severity at 4800 m (3 days later), whereas Lake Louise scores from the same time points were unrelated. These findings highlight the potential for pulse oximetry recorded from lower camps to be predictive of acute mountain sickness severity at higher altitude.

## 1 INTRODUCTION

Acute mountain sickness (AMS) is one of three major high‐altitude illnesses (including high‐altitude cerebral and pulmonary oedema; Imray et al., 2011) and afflicts between 35% and 75% of the hundreds of thousands of individuals ascending to high altitude (>2500 m a.s.l.) each year (Croughs et al., 2014; Karinen et al., 2008). The defining clinical feature of AMS is headache, which, along with other symptoms of gastrointestinal distress, fatigue and dizziness/light‐headedness, is scored subjectively using the Lake Louise score (LLS) criteria (range: 0–3) (Roach et al., 2018). Although AMS is an obvious diagnosis in its severe form, the early stages of AMS are poorly defined and easily confused with other illnesses. This subjectivity of LLS, together with being able to track illness only as symptoms develop, makes prospective assessments and predictions for AMS impossible (Moore et al., 2020). Therefore, objective physiological measurements have been pursued as predictors of AMS.

The hypoxaemic challenge associated with high altitude is the obvious underlying cause of AMS and would therefore suggest that measurement of arterial oxygenation should be helpful for predicting those most at risk for AMS and detecting those who are worst affected. However, the measurement of arterial blood gases, which is the gold standard for measuring hypoxaemia in clinical medicine, in series (i.e., daily) is rare at high altitude owing to the associated increase in risks imposed by and challenges surrounding collection and analysis of biological specimens in austere environments. A non‐invasive alternative to arterial blood gases is the measurement of peripheral oxygen saturation ($S_{pO_{2}}$) by pulse oximetry, which is often used by medical practitioners during trekking/climbing expeditions to monitor unwell individuals or patients with pre‐existing conditions.

The use of pulse oximetry has also become increasingly common among guides, trekkers and climbers to monitor members of their group (Luks & Swenson, 2011). Nevertheless, the literature surrounding the utility of pulse oximetry at altitude has produced conflicting results, which both support (Burtscher et al., 2019) and negate (Chen et al., 2012; Jun‐Bo et al., 2016; Leichtfried et al., 2016; O'Connor et al., 2004; Wagner et al., 2012) the use of $S_{pO_{2}}$ as a predictor/detector of AMS. Inconsistencies in the literature might be attributable to differing methodologies, such as type/quality of oximeters used and the time of day (e.g., morning vs. overnight) and/or duration of measurements (e.g., 60 s vs. continuous) (Luks & Swenson, 2011). Therefore, it is clear that further investigation is required for the optimization of $S_{pO_{2}}$ measurement methodology in the context of its utility at high altitude and potential use as a possible predictor of AMS.

Continuous overnight pulse oximetry might be more advantageous than one‐off daytime measurements for several reasons. These include reduced movement artefact and light interference, in addition to issues with cold fingers, all of which increase the vulnerability to inaccuracy of the $S_{pO_{2}}$ measurement (Luks & Swenson, 2011). Likewise, given that the greatest hypoxaemic insult occurs overnight at altitude, it would be reasonable to suggest that measurement of desaturation severity overnight might be a key factor when trying to predict those who are at greatest risk for developing AMS (Matsuzawa et al., 1994).

The overall objective of this observational study was to quantify the altitude‐induced hypoxaemia in individuals during an ascent to 4800 m a.s.l. to determine the utility of $S_{pO_{2}}$ measurement to predict AMS outcomes. Specifically, we aimed to evaluate high‐altitude pulse oximetry $S_{pO_{2}}$ measurements in the context of the following factors: (1) standardized measurements [i.e., arterial oxygenation ($S_{aO_{2}}$)]; (2) collection methodology (e.g., morning spot measures vs. continuous overnight measures); and (3) AMS symptomology. In addition, we aimed to present large time‐series data (i.e., overnight $S_{pO_{2}}$) in a way that could be interpreted easily for clinical decision‐making. It was hypothesized that: (1) overnight $S_{pO_{2}}$ (ov‐$S_{pO_{2}}$), as opposed to morning $S_{pO_{2}}$ (mo‐$S_{pO_{2}}$), would more accurately reflect $S_{aO_{2}}$ at high altitude; and (2) ov‐$S_{pO_{2}}$ would be a better indicator/predictor of AMS during ascent compared with brief morning measurements, owing to the increased information that can be extracted from overnight recordings.

## 2 MATERIALS AND METHODS

### 2.1 Ethical approval

Ethical approval was granted by the University of Birmingham (ERN_19‐0325). The study conformed to the standards set by the *Declaration of Helsinki* 2018, except for registration in a database. Methodology was reported in accordance with the STROBE guidelines for observational studies, in addition to the STAR data reporting guidelines, which are specific to high‐altitude research (Brodmann Maeder et al., 2018). Statistical power/sample size was not calculated prospectively owing to group size restrictions imposed by the guiding team, which ultimately resulted in a maximum of 20 possible participants. Written informed consent was obtained from all participants prior to participation.

### 2.2 Participants and design

**FIGURE 1:**
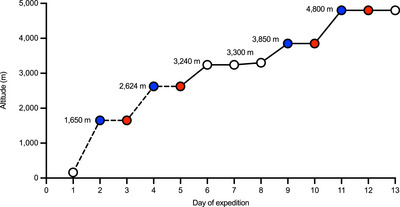
Expedition profile. The study involved the ascent to a top camp at 4800 m in the northeastern Indian Himalaya near the Zema Glacier at the base of Kanchenjunga over a period of 13 days. To day 5, the ascent was primarily motorized (dashed line); from day 6 onwards it was on foot (continuous line). Arterialized capillary samples were collected as indicated by red circles, with these data being compared with morning oxygenation (mo‐$S_{pO_{2}}$) from the same morning and overnight recordings that were initiated the night before (marked by blue circles), albeit completed on the same morning.

Participants were recruited from members of the Birmingham Medical Research Expeditionary Society who planned to embark on a high‐altitude expedition to Sikkim (northeast Indian Himalaya). Recruited participants were healthy, non‐smoking lowlanders between the ages of 18 and 80 years. Baseline characteristics (i.e., age, height, body mass and history of altitude illness during ascent to >3500 m a.s.l.) were recorded in the UK (∼140 m a.s.l.) within the 3 weeks prior to departure for Sikkim. Unacclimatized participants then ascended to high altitude in accordance with Figure 1 and without the prophylactic use of pharmacological agents known to improve oxygenation (e.g., acetazolamide) (Joyce et al., 2018), unless prescribed by expedition medical officers owing to the severity of AMS symptoms. During ascent, mountain sickness scores, arterialized capillary samples and peripheral oxygenation measurements (morning and overnight) were collected and analysed upon return to the UK as described below.

### 2.3 Mountain sickness scores

Lake Louise score criteria (Roach et al., 2018) were recorded each morning and evening during the ascent to track AMS symptoms. On transit days (refer to Table 1), evening scores were completed ∼6 h after arriving at camp, with morning scores obtained ∼8–10 h later (i.e., the following morning). Morning scores were used to categorize individuals as AMS‐positive (AMS^+^; total LLS ≥ 3, with headache ≥ 1) or AMS‐negative (AMS^−^; total LLS < 3, without headache) for the purposes of subgroup analysis of saturation data.

**TABLE 1:** Nocturnal pulse oximetry features compared between individuals scoring positive or negative for acute mountain sickness.

Parameter	AMS^+^	AMS^−^	Median difference [95% CIs]	*P*‐value
Descriptive statistics				
mo‐$S_{pO_{2}}$ (%)	85 [81–86]	90 [86–93]	−5.0 [−8.0 to −3.0]	<0.001*
ov‐$S_{pO_{2}}$ (%)	81 [77–86]	86 [81–90]	−5.2 [−7.6 to −1.7]	0.002*
HR (beats min^−1^)	63 [58–75]	60 [55–67]	4.5 [0.9–11.3]	0.016*
HR/$S_{pO_{2}}$ (beats min^−1^ %^−1^)	0.82 [0.69–0.97]	0.70 [0.63–0.81]	0.12 [0.03–0.19]	<0.001*
Δ$S_{pO_{2}}$ (%)	0.07 [−0.46 to 0.51]	0.20 [−0.13 to 0.53]	−0.19 [−0.53 to 0.12]	0.295

Statistical moments				
Kurtosis proper	3.89 [3.42–4.44]	4.15 [3.41–5.10]	−0.28 [−0.75 to 0.25]	0.333
Skewness	0.28 [0.14–0.53]	0.30 [0.01–0.59]	−0.15 [−0.15 to 0.21]	0.679
Variance	6.17 [2.89–8.90]	2.72 [1.82–4.54]	3.45 [0.87–4.63]	0.001*

Desaturation characteristics				
Total number of desaturations	336 [75–598]	75 [37–187]	261 [48–381]	<0.001*
Duration of desaturation (s)	18.1 [15.5–24.2]	23.4 [19.5–30.3]	−5.3 [−7.9 to −1.8]	0.002*
ODI (desaturations h^−1^)	43.2 [9.2–67.1]	9.1 [5.2–20.0]	34.1 [6.4–42.2]	<0.001*
Hypoxic burden (% min h^−1^)	168.1 [134.2–206.1]	111.1 [69.8–161.1]	57.1 [37.2–90.5]	<0.001*
TST80 (%)	60.9 [4.4–95.3]	0.15 [0.0–25.0]	69.7 [11.0–70.0]	<0.001*

*Note*: Results are presented as the median [25%–75% quartiles] for all variables for both subgroups. Data were categorized as positive or negative for acute mountain sickness (AMS^+^ vs. AMS^−^ subgroups, respectively) based on the Lake Louise score on the following morning using a priori diagnostic cut‐off > 3 with at least one point for headache. Data were then compared between AMS^+^ and AMS^–^ subgroups using Mann–Whitney *U*‐test with significance set to α < 0.05. Effect sizes between subgroups are presented as the median differences (95% confidence intervals). Δ$S_{pO_{2}}$ represents the difference between means from the second and first halves of the night (i.e., second half mean minus first half mean). * denote significant *p*‐value (i.e., α < 0.05).

Abbreviations: AMS, acute mountain sickness; CI, confidence interval; ODI, oxygen desaturation index; $S_{pO_{2}}$, peripheral oxygen saturation; TST80, total saturation time spent at <80% $S_{pO_{2}}$.

### 2.4 Arterialized capillary oxygen saturation

Arterialized capillary blood samples, a less invasive alternative to traditional arterial blood gases, with serial earlobe sampling being feasible at high altitude (Lewis et al., 2018), were collected at baseline and during the mornings (∼60–90 min after waking) of days 3, 5, 10 and 12 during ascent, which followed an increase in sleeping altitude (Figure 1, red circles). Samples were obtained from the earlobe after warming for several minutes with a hot water bottle, as previously described (Lewis et al., 2018; Nawrocki et al., 2021), then analysed immediately using the i‐STAT point‐of‐care blood gas analyser and EG6+ cartridges (Abbott Laboratories, Chicago, IL, USA). The resultant arterialized capillary oxygen saturation ($S_{aO_{2}}$) measurements were used as a ‘standard’ measure of blood oxygen saturation, against which the mo‐$S_{pO_{2}}$ measurements (collected the same morning) and ov‐$S_{pO_{2}}$ (from the preceding night) were validated (refer to Figure 1, blue vs. red circles).

### 2.5 Pulse oximetry

The $S_{pO_{2}}$ and heart rate (HR) were measured from the finger pulp using portable wrist‐worn pulse oximeters (WristOx_2_, 3150 model; Nonin Medical, Plymouth, MN, USA; reliability: ±2%–3% $S_{pO_{2}}$ and 3–4 beats) and accompanying PureLight flexible sensors (8000J, Nonin Medical), both in the morning (mo‐$S_{pO_{2}}$) and overnight (ov‐$S_{pO_{2}}$).

#### 2.5.1 Morning pulse oximetry recordings

The Mo‐$S_{pO_{2}}$ measurements were collected by expedition medical officers while participants were seated during routine morning medical examinations (days 2–13). These were conducted during regular morning activities (e.g., breakfast, packing) and when participants had been upright and out of their tents for ≥30 min. Medical officers were instructed to average $S_{pO_{2}}$ readings intuitively from the display of the oximeter over ∼60–90 s (or until the measurement stabilized), as previously described (Tannheimer & Lechner, 2019).

#### 2.5.2 Overnight pulse oximetry recordings

##### Collection and preprocessing

**FIGURE 2:**
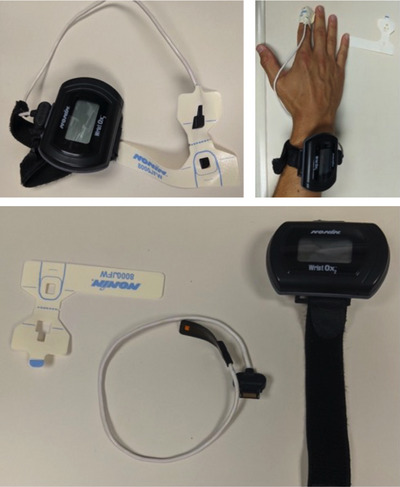
Pulse oximeters. The WristOx 3150 model (Nonin Medical) pulse oximeter is pictured alongside the adult flexible finger sensor (8000J) and accompanying disposable adhesive fastener. Photograph credit(s) Drs Johnson and Wheatley‐Guy, and Mr Jesse Schwarz of the Johnson Lab, Mayo Clinic, Scottsdale campus in Scottsdale, AZ, USA.

Before baseline ov‐$S_{pO_{2}}$ data collection, participants were provided with the manufacturer‐supplied instructional video demonstrating sensor placement and securement (with adhesive finger wraps, see Figure 2) and written instructions, which detailed device data collection procedures. For all recordings, participants were asked to: (1) plug the sensor in to the oximeter (to initiate the recording) after it was secured and immediately before bed (i.e., once they were lying down and intended to sleep); and (2) immediately unplug the sensor from the oximeter (to end the recording) upon waking and getting out of bed to start their day.

Participants wore the oximeter for two nights in the UK for familiarization and baseline measures, then on days 2–13 during ascent. Data were recorded continuously (1 Hz) and offloaded with nVision software (v.6.5.1; Nonin Medical), processed (i.e., artefact removal) and saved in an offline electronic database until analysis (upon return to the UK). Note that data artefacts were identified, counted and removed using a bespoke MATLAB program (v.2022a; MathWorks, Natick, MA, USA) based on the following criteria: (1) $S_{pO_{2}}$ value <30%; (2) arbitrary $S_{pO_{2}}$ value >100% (e.g., 500% for WristOx_2_ devices); or (3) deviation in $S_{pO_{2}}$ of >4% from the preceding second (Taha et al., 1997). The artefact index (AI), or the percentage of the total raw overnight recording identified as artefact, was estimated and reported to ensure quality of recordings.

##### Feature extraction and visualization of overnight $S_{pO_{2}}$ (ov‐$S_{pO_{2}}$) recordings

The mean ± SD was calculated for overnight measures of $S_{pO_{2}}$, HR and HR/$S_{pO_{2}}$ ratio (in beats per minute per percentage). Additional features extracted from overnight recordings included: change in $S_{pO_{2}}$ ($\Delta S_{pO_{2}}$), calculated as the difference between averages from the first to second halves of sleep duration (Tannheimer et al., 2017), total number of desaturations, desaturation duration, desaturation frequency [or oxygen desaturation index (ODI), expressed as the number of desaturations per hour] and hypoxic burden (or desaturation area; Azarbarzin et al., 2019; Kulkas et al., 2015). Desaturations were identified using previously published criteria (Taha et al., 1997), albeit with an altitude‐corrected $S_{pO_{2}}$ nadir (≤4% of the preceding 20 s), as described by Cross et al. (2015).

For the investigation of multiple overnight datasets simultaneously (e.g., to compare between individuals or within individuals between nights), cumulative and relative frequency plots were constructed from artefact‐free datasets (Harigopal et al., 2011; Terrill, 2019; Terrill et al., 2018, 2015). Total or cumulative sleep time (TST or CT, respectively) spent at or below a specific $S_{pO_{2}}$ threshold (e.g., TST of <80% $S_{pO_{2}}$), which has previously been extracted at altitude (Cross et al., 2015), was estimated to describe cumulative frequency plots quantitatively.

Likewise, to describe ov‐$S_{pO_{2}}$ relative frequency plots, statistical moments, such as the arithmetic mean, variance, skewness and kurtosis proper, were estimated as previously described (Harigopal et al., 2011; Terrill, 2019; Terrill et al., 2018, 2015). The ov‐$S_{pO_{2}}$ variance represented the overall ‘fluctuation’ in peripheral oxygenation overnight. Kurtosis, in this case, reflected the ‘peakedness’ (or ‘flatness’) of ov‐$S_{pO_{2}}$ data, with higher values reflecting greater ‘peakedness’ on visual inspection, which indicated a greater magnitude of time spent at a particular saturation. In healthy individuals at sea level, a high kurtosis would be expected, because most of the night is spent at a select few saturations (e.g., 97%–100% $S_{pO_{2}}$). In contrast, at high altitude, a lower kurtosis would be expected, because the time throughout the night is likely to be spread across a wider range of saturations (‘flatter’ distribution). Owing to the large number of data points collected overnight, a ‘significant’ kurtosis (proper) was considered to be a value of >7 (Kim, 2013; West et al., 1995). The ov‐$S_{pO_{2}}$ skewness described the relationship between the mean and median of overnight data. Negativity or positivity was indicative of the directional inflection of the peak and the location of the mean relative to the remaining portion of ov‐$S_{pO_{2}}$ data. More specifically, positive skew indicated that the ‘tail’ on the right side of the ‘peak’ was longer than the one on the left and that the majority of the ov‐$S_{pO_{2}}$ data being analysed were located to the left of the mean (Kim, 2013). In contrast, negative skew indicated that the ‘tail’ on the left side was longer than that on the right and that a majority of the ov‐$S_{pO_{2}}$ data being analysed were located to the right of the mean (Kim, 2013). An absolute skew of ≥2 was used to identify substantial asymmetry in the data (Kim, 2013; West et al., 1995).

##### Exploratory features for potential clinical application

In addition to the mean ± SD outlined above, moving averages were applied (i.e., 60, 15 and 3 min; 30 s) to artefact‐free overnight recordings, with the lowest ov‐$S_{pO_{2}}$ moving average extracted for each of the indicated averaging windows. Likewise, the beginnings and ends of the overnight recordings were investigated further, with 15 min sections of the averaged data extracted for comparison with AMS outcomes at 4800 m. In addition, mo‐$S_{pO_{2}}$ and ov‐$S_{pO_{2}}$ data collected from 3300 to 4800 m were also compared with AMS outcomes (at both 4800 and 3850 m) to explore how predictive recordings from lower altitude were for peak AMS.

### 2.6 Statistical analysis

Normality (or lognormality) of distribution was assessed for nightly group data for all features, with any outliers (±3 SDs) removed prior to analysis. Descriptive statistics (e.g., mean ± SD) were reported for daily/nightly group data unless otherwise specified. Statistical analyses were performed using Prism (v.8.3.0 for Mac iOS; GraphPad Software, San Diego, CA, USA) and MATLAB v.2022a (MathWorks). All statistical tests were two tailed, with significance set to α < 0.05, unless otherwise specified.

#### 2.6.1 $S_{aO_{2}}$ versus peripheral oxygen saturation (ov‐$S_{pO_{2}}$ or mo‐$S_{pO_{2}}$)

A priori hypotheses to be tested were the agreements between $S_{aO_{2}}$ and ov‐$S_{pO_{2}}$ or mo‐$S_{pO_{2}}$ (independently) and between ov‐$S_{pO_{2}}$ and mo‐$S_{pO_{2}}$. Comparisons between $S_{aO_{2}}$ and ov‐$S_{pO_{2}}$ or $S_{aO_{2}}$ and mo‐$S_{pO_{2}}$ measurements were facilitated utilising Bland–Altman plots (Altman & Bland, 1983; Bland & Altman, 1995) (plotted as the differences between $S_{aO_{2}}$ and ov‐$S_{pO_{2}}$ or mo‐$S_{pO_{2}}$ measurements versus the averages) (Chhapola et al., 2015). The Bland–Altman plot for ov‐$S_{pO_{2}}$ versus mo‐$S_{pO_{2}}$ measurements incorporated only pairs of measurements that had been validated against corresponding $S_{aO_{2}}$ measurements. Bias and agreement (95% limits of agreement) were estimated for all Bland–Altman plots, with the best‐fitting lines of the simple linear regressions plotted and the slopes, *x*‐intercepts and goodness of fits reported. Last, mixed‐effects analysis with Tukey's *post hoc* test was used to make comparisons between oxygenation measurements (e.g., $S_{aO_{2}}$ vs. ov‐$S_{pO_{2}}$ vs. mo‐$S_{pO_{2}}$) and time points of interest (e.g., day 2 vs. day 11).

#### 2.6.2 Subgroup analysis

Subgroup analysis was performed between AMS^+^ and AMS^−^ individuals to evaluate the predictability of illness by features (i.e., to investigate whether features exhibited any clinical significance). Subgroup data were presented as the median ± interquartile ranges (IQRs) (from across the expedition) and compared using the Mann–Whitney *U*‐test. Median differences [95% confidence intervals (CIs)] were also presented for subgroups. For features exhibiting a significant difference between AMS^+^ versus AMS^–^, the receiver operating characteristic (ROC; Wilson/Brown method) (Hajian‐Tilaki, 2013) was conducted as previously described (Chawla & Tripathi, 2015), with the area under the curve (AUC; minimum set to chance level, 0.50) used to assess diagnostic accuracy (Hanley & McNeil, 1982; Kumar & Indrayan, 2011). Youden's index was used to identify optimal cut‐off values (or best operating points) for detection of AMS, with corresponding sensitivity and specificity reported.

#### 2.6.3 Exploratory analyses for potential clinical application

Pearson's correlation (*r*) was used to evaluate relationships between exploratory features and LLS. Specifically, correlation analysis was performed between ov‐$S_{pO_{2}}$ measurements (from 3300 to 4800 m) and the LLS from the morning on which the highest symptom scores were reported to evaluate the utility of ov‐$S_{pO_{2}}$ as a potential biomarker of AMS outcome. For comparison, correlation analysis was also performed between mo‐$S_{pO_{2}}$ measurements from the corresponding mornings (following overnight measurements) and the LLS from the morning on which the highest symptom scores were reported.

## 3 RESULTS

### 3.1 Participant characteristics

Eighteen (7 females and 11 males) were included in this study. Baseline characteristics were as follows: age, 36 ± 16 years; height, 175 ± 11 cm; and body mass, 72.2 ± 11.6 kg. One individual reported a history of severe high‐altitude illness (i.e., high‐altitude cerebral or pulmonary oedema) during a past ascent, and three separate individuals reported a history of AMS during previous high‐altitude ascents, with all these reported previous illnesses occurring >2 years prior to this study.

### 3.2 Mountain sickness scores

**FIGURE 3:**
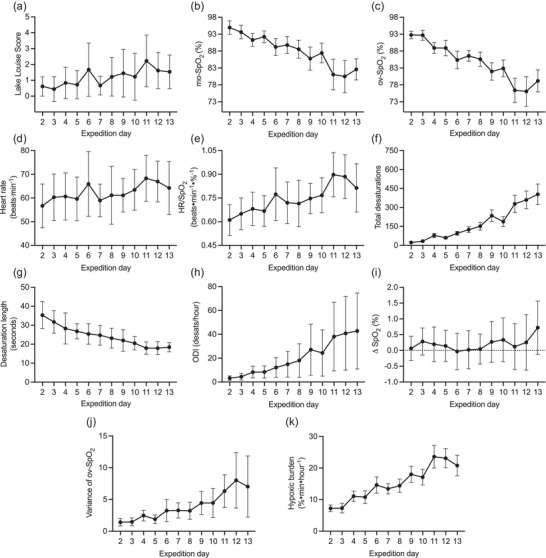
(a) Daily Lake Louise scores. (b) Morning oxygen saturation (mo‐$S_{pO_{2}}$). (c) Overnight oxygen saturation (ov‐$S_{pO_{2}}$). (d) Overnight heart rate (HR). (e) Overnight heartrate‐to‐saturation ratio (HR/$S_{pO_{2}}$). (f) Total number of oxygen desaturations. (g) Desaturation duration. (h) Oxygen desaturation index (ODI; in desaturations per hour). (i) Percentage change in oxygen saturation. (j) Variance of overnight oxygen saturation. (k) Overnight hypoxic burden.

Morning LLS are presented in Figure 3a, with the greatest symptom burden (LLS_peak_, 2 ± 2) observed on the first morning at 4800 m (day 11). Three individuals were treated with acetazolamide because they developed moderate‐to‐severe AMS symptoms (LLS score of ≥5).

### 3.3 Arterial(ized) capillary oxygen saturation

From the 72 possible arterialized capillary blood collections during ascent (on 4 days), 67 samples were collected and analysed successfully.

#### 3.3.1 $S_{aO_{2}}$ versus mo‐$S_{pO_{2}}$

**FIGURE 4:**
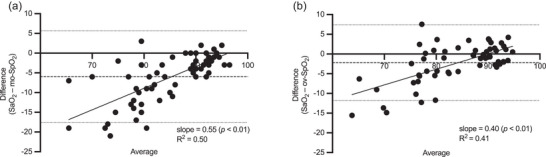
Bland–Altman plots for comparisons between arterialized capillary oxygenation ($S_{aO_{2}}$) and morning (a) and overnight (b) peripheral oxygenation (mo‐$S_{pO_{2}}$ and ov‐$S_{pO_{2}}$, respectively). Slopes and coefficients of determination are presented for the best‐fitting lines that were plotted from the linear regressions. Average bias is represented by a dashed line, and the 95% limits of agreement are represented by dotted lines.

A Bland–Altman plot comparing $S_{aO_{2}}$ and mo‐$S_{pO_{2}}$ was constructed from 67 pairs of measurements (Figure 4a) and had a bias of −6.0% ± 5.9% saturation and 95% limits of agreement from −17.6% to 5.7% saturation. The slope of the best‐fitting line plotted from the simple regression deviated significantly from zero (0.55 [CIs: 0.41 to 0.68]; *p *< 0.01), while the *x*‐intercept of the best‐fit‐line was 96.5% (CIs: 94.2% to 99.9%).

#### 3.3.2 $S_{aO_{2}}$ versus ov‐$S_{pO_{2}}$

A Bland–Altman plot comparing $S_{aO_{2}}$ and ov‐$S_{pO_{2}}$ was constructed from 59 pairs of measurements (Figure 4b) and was observed to have lower bias −2.2% ± 4.9% saturation and 95% limits of agreement (−11.8% to 7.4% saturation) compared with mo‐$S_{pO_{2}}$ comparisons. Similar to the $S_{aO_{2}}$ versus mo‐$S_{pO_{2}}$ data, the slope of the best‐fitting line plotted for $S_{aO_{2}}$ versus ov‐$S_{pO_{2}}$ Bland–Altman data deviated significantly from zero (0.40 [CIs: 0.27 to 0.52]; *P *< 0.01), and the *x*‐intercept of the best‐fitting line was 89.8% (CIs: 87.1% to 93.7%), hence lower than that for $S_{aO_{2}}$ versus mo‐$S_{pO_{2}}$.

### 3.4 Peripheral oxygen saturation

#### 3.4.1 Morning pulse oximetry measurements

Ninety‐nine per cent of possible mo‐$S_{pO_{2}}$ measurements were collected and analysed successfully. Daily mo‐$S_{pO_{2}}$ (mean ± SD) data are presented in Figure 3b.

#### 3.4.2 Overnight pulse oximetry recordings

Ninety per cent of possible overnight recordings were collected and analysed successfully, with the mean artefact index of these recordings being very low (0.75% ± 1.5%). The mean artefact‐free recording duration was 7.97 ± 1.23 h. Nightly results estimated from artefact‐free recordings for ov‐$S_{pO_{2}}$, HR, HR/ov‐$S_{pO_{2}}$, total desaturations, desaturation length, ODI and hypoxic burden are presented in Figure 3c–k.

Heart rate (68 ± 11 beats min^−1^) and HR/ov‐$S_{pO_{2}}$ (0.90 ± 0.14 beats min^−1^ %^−1^) were highest during the first night at 4800 m (day 11). Mean ov‐$S_{pO_{2}}$ was lowest (76% ± 4%), hypoxic burden highest (23.6% min h^−1^ ± 3.6% min h^−1^) and desaturation duration shortest (17.9 ± 3.5 s) during the second night at 4800 m (day 12). The total number of desaturations (404 ± 307), ODI (42.7 ± 31.9 desaturations h^−1^) and $\Delta S_{pO_{2}}$ (0.72% ± 0.84%) were highest during the third and final night at 4800 m (day 13).

**FIGURE 5:**
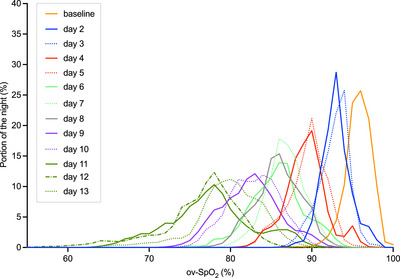
Averaged relative frequency distributions for overnight peripheral oxygenation (ov‐$S_{pO_{2}}$) recordings during ascent. Cumulative and relative frequency plots were constructed successfully for all nocturnal recordings. Data were plotted as the mean relative frequency (of the group) for each $S_{pO_{2}}$ between 30% and 100%. The first night at a given camp is denoted with a continuous line, with the second night in the same colour with a dotted line and third night with a dashed line. Kurtosis proper (or ‘peakedness’) was reduced with increasing altitude, representing a wider distribution of $S_{pO_{2}}$ values (a ‘flattening’ of the frequency distribution).

Relative frequencies were constructed successfully for each successful overnight recording, with an averaged representation from the group presented for conceptualization purposes (Figure 5). Examination of these relative frequency data showed that kurtosis proper was lowest (or ‘flattest’) during the second night at 4800 m (3.44 ± 0.88), and improved (i.e., value increased) with partial acclimatization, which was most evident in the comparisons between back‐to‐back nights at the same altitude (e.g., day 6 vs. 7, continuous vs. dashed lines). Likewise, skewness of ov‐$S_{pO_{2}}$ was negative at baseline (−0.31 ± 0.85), positive each night of ascent, and greatest (0.40 ± 0.51) during the first night at 3850 m (day 9). Finally, ov‐$S_{pO_{2}}$ variance was greatest (6.3 ± 2.6) during the second night at 4800 m (day 12) (Figure 3k).

### 3.5 Subgroup analysis

Results from Mann–Whitney *U*‐test for comparisons between AMS^+^ and AMS^−^ subgroups for mo‐$S_{pO_{2}}$ and ov‐$S_{pO_{2}}$ and extracted features are presented in Table 1. The AUCs are presented in Table 2 alongside optimal cut‐off values and corresponding sensitivity and specificity values from the ROC plots. Differences between AMS^–^ and AMS^+^ were highlighted further by the comparisons of relative and cumulative frequency plots between two individuals (one AMS^–^ and one AMS^+^), as shown in Figure S1A.

**TABLE 2:** Results from receiver operating characteristic plots for the classification of individuals as positive or negative for acute mountain sickness using pulse oximetry.

Parameter	AUC	95% CIs	*P*‐value	Cut‐off value	Sensitivity (%)	Specificity (%)	Youden's index
mo‐$S_{pO_{2}}$ (%)	0.770	0.676–0.863	<0.001	<85.5%	66.7	76.0	1.427
ov‐$S_{pO_{2}}$ (%)	0.796	0.699–0.879	<0.001	<82.7%	79.0	70.8	1.497
HR/ov‐$S_{pO_{2}}$ (beats min^−1^ %^−1^)	0.694	0.573–0.815	0.006	>0.817	57.9	76.0	1.406
Variance	0.726	0.598–0.855	0.001	>6.056	58.0	87.2	1.451
Total overnight desaturations	0.753	0.637–0.868	<0.001	>285.0	57.9	85.4	1.433
Desaturation duration (s)	0.716	0.582–0.851	0.002	<18.3	57.9	82.6	1.405
Oxygen desaturation index (desaturations h^−1^)	0.756	0.637–0.875	<0.001	>34.7	57.9	86.8	1.447
Hypoxic burden (% min h^−1^)	0.798	0.708–0.888	<0.001	>119.6	94.7	57.6	1.523
TST80 (%)	0.782	0.676–0.889	<0.001	>2.07	80.0	66.3	1.499

*Note*: The ROC plots for both morning (mo‐$S_{pO_{2}}$) and overnight (ov‐$S_{pO_{2}}$) oxygen saturation are included, along with additional metrics derived from ov‐$S_{pO_{2}}$ only.

Abbreviations: AUC, area under the curve; 95% CIs, 95% confidence intervals of the AUC; ROC, receiver operating characteristic; $S_{pO_{2}}$, peripheral oxygen saturation; TST80, total saturation time spent below 80% $S_{pO_{2}}$.

#### 3.5.1 Exploratory analyses for potential clinical application

Table 3 presents all results from exploratory analysis related to $S_{pO_{2}}$ (morning and overnight). The key significant observations were as follows: (1) ov‐$S_{pO_{2}}$ data from the first night at 3850 m were significantly correlated with LLS_peak_ at 4800 m compared with lower altitude (3300 m) and the second night at 3850 m, which was consistent across all time intervals and portions of data analysis (e.g., lowest 60 min average vs. first or last 15 min portion); and (2) all first night data at 4800 m were correlated with LLS the next morning, when AMS symptoms were highest (LLS_peak_). In contrast, there was no significant correlation between LLS_peak_ and mo‐$S_{pO_{2}}$ from 3300 or 3850 m. The mo‐$S_{pO_{2}}$ was correlated with LLS_peak_ only when collected on the same morning (i.e., at 4800 m). Likewise, there was no significant correlation between LLS_peak_ and LLS from the first morning at 3850 m, although LLS_peak_ was correlated with LLS from 3300 m and the second morning at 3850 m (refer to Table 3).

**TABLE 3:** Results from Pearson correlation for exploratory analyses of pulse oximetry data.

Parameter	3300 m (night −3)		First night at 3850 m (night −2)			Second night at 3850 m (night −1)		First night at 4800 m (night 0)	
	***r***	***P*‐value**	***r***	***P*‐value**		***r***	***P*‐value**	***r***	***P*‐value**
LLS_peak_ (4800 m) vs. $S_{pO_{2}}$ from:									
mo‐$S_{pO_{2}}$	−0.275	0.285	−0.224	0.388		−0.410	0.102	−0.584	0.014
ov‐$S_{pO_{2}}$									
Lowest 60 min average overall	−0.155	0.596	−0.585	0.028*		−0.380	0.146	−0.656	0.011*
Lowest 30 min average overall	−0.118	0.688	−0.589	0.027*		−0.359	0.172	−0.625	0.017*
Lowest 15 min average overall	−0.080	0.783	−0.602	0.023*		−0.354	0.178	−0.615	0.019*
First 15 min average	−0.243	0.403	−0.614	0.020*		−0.470	0.066	−0.555	0.040*
Last 15 min average	0.010	0.988	−0.582	0.029*		−0.184	0.495	−0.763	0.002*
LLS_peak_ vs. LLS from days nights prior									
LLS_peak_ vs. LLS from:	0.599	0.014*	0.457		0.076	−0.609	0.012*	n.a.	n.a.

*Note*: Overnight oxygen saturation (ov‐$S_{pO_{2}}$) data were computationally averaged increments of 15 min, with the first and last 15 min of the recordings also assessed. These overnight $S_{pO_{2}}$ measurements, in addition to morning $S_{pO_{2}}$ measurements (mo‐$S_{pO_{2}}$), were analysed further alongside the Lake Louise scores (LLS) from the highest camp (4800 m) when symptoms were greatest (LLS_peak_), with the LLS from lower altitudes also compared with LLS_peak_. * denote significant *p*‐value (i.e., α < 0.05).

## 4 DISCUSSION

The objective of this observational study was to quantify the altitude‐induced hypoxaemia in individuals during an ascent to 4800 m to determine the utility of nocturnal pulse oximetry measures for the prediction of AMS. The main findings were as follows: (1) ov‐$S_{pO_{2}}$ parameters were significantly different between AMS^+^ and AMS^−^; (2) ov‐$S_{pO_{2}}$ cumulative and frequency plots were useful for visualization of acclimatization status; and (3) exploratory analysis revealed significant relationships between overnight oximetry data and LLS_peak_, although the same was not observed between mo‐$S_{pO_{2}}$ and LLS_peak_. Collectively, these findings highlight the potential for overnight pulse oximetry measures to be used to predict AMS‐susceptible individuals as they ascend to high altitude.

### 4.1 Utility of nocturnal pulse oximetry measures for predicting AMS

Very few instances outside of infectious disease have a diagnostic marker that can be isolated as the cause of disease. Oxygen levels in AMS, however, might be one of these rare diagnostic markers. Here, we show that the relationship between ov‐$S_{pO_{2}}$ and LLS observed is not only consistent with previous studies (Burgess et al., 2004), but that it can be used to differentiate between those with/at risk for developing AMS using the additional methods and metrics described herein, which provides support for the use of overnight $S_{pO_{2}}$ in tracking and predicting AMS susceptibility.

The present study demonstrated that ov‐$S_{pO_{2}}$, compared with mo‐$S_{pO_{2}}$, was more consistent with $S_{aO_{2}}$ measurements and measured lower levels of oxygenation. The reasoning for this is likely to be twofold. First, pulse oximetry in general is known to underestimate $S_{aO_{2}}$; however, oxygenation is lower overnight at altitude, which would suggest that overnight measurements would more closely mirror our daytime $S_{aO_{2}}$ measure. Second, $S_{pO_{2}}$ is lower when measured supine than when seated upright (Kuenzel et al., 2020). Taken together, these factors provide a likely explanation for why $S_{pO_{2}}$ measurements obtained overnight were more consistent with the $S_{aO_{2}}$ measurements obtained while seated upright in the morning.

Further emphasis for the advantage of ov‐$S_{pO_{2}}$ compared with mo‐$S_{pO_{2}}$ for its predictive utility during ascent to altitude was illustrated by the results from ROCs and from our exploratory analyses. For example, although the median differences between AMS subgroups were similar between mo‐$S_{pO_{2}}$ and ov‐$S_{pO_{2}}$ (both ∼5% lower in the AMS^+^ subgroup; Table 1), albeit a measured lower cut‐off value with ov‐$S_{pO_{2}}$ (83% vs. 86%; Table 2), the corresponding sensitivity was higher for the ov‐$S_{pO_{2}}$ cut‐off value (79.0% vs. 66.7%; see Table 2), which is arguably more important in the context of predicting AMS. This minor difference is strengthened by results from our exploratory analysis, which demonstrated that ov‐$S_{pO_{2}}$ and not mo‐$S_{pO_{2}}$ obtained days prior to the onset of peak symptoms was correlated with LLS when symptom burden was greatest (at 4800 m). Note that mo‐$S_{pO_{2}}$ was correlated with LLS only when collected on the same morning (i.e., at 4800 m), making it more diagnostic than predictive of AMS at this time point of ascent.

Extraction of additional metrics by overnight pulse oximetry recordings further increases the value of overnight oximetry over and above that of morning one‐off measurements, despite the ease of punctual mo‐$S_{pO_{2}}$ measurements. In comparison to one‐off measurements typically made during high‐altitude expeditions [e.g., ≤15 min (Mandolesi et al., 2014)], overnight oximetry affords the benefit of capturing additional vital information that might be relevant to the acclimatization status (Ainslie et al., 2013) or the progression of high‐altitude illnesses (Erba et al., 2004; Nespoulet et al., 2012) [e.g., apnoea‐related events (Marcos et al., 2009; Terrill, 2019); relative change in $S_{pO_{2}}$ during the night (Tannheimer et al., 2017); TST < 80% (Cross et al., 2015; Mandolesi et al., 2014); ODI (Burgess et al., 2004; Erba et al., 2004)]. Our findings add to this previous work, with the cumulative and relative frequency plots of the overnight oximetry data created using the bespoke MATLAB program offering a unique advantage, enabling the comparison of multiple nocturnal recordings and metrics (skewness, kurtosis and variance) simultaneously (and/or across individuals simultaneously), which is not currently possible using the existing manufacturer's software (i.e., nVision). Moreover, frequency plots for $S_{pO_{2}}$ successfully demonstrated changes as a result of partial acclimatization, which was most evident from comparisons between back‐to‐back nights at the same camp (see Figure 5 and Figure S1A). Likewise, relative frequency plots (from which skewness and kurtosis proper are derived) were useful for distinguishing between AMS^+^ (Figure S1A) and AMS^−^ (Figure S1B) individuals. Furthermore, attributes of cumulative frequency plots (i.e., TST < 80% $S_{pO_{2}}$) support previous findings that have shown $S_{pO_{2}}$ to be a clinically meaningful metric with regard to AMS symptomology (Mandolesi et al., 2014).

There were significant differences between AMS^+^ and AMS^−^ individuals for mean nocturnal $S_{pO_{2}}$. These findings are consistent with those from Burtscher et al. (2019), in addition to Karinen et al. (2010) and Koehle et al. (2010), all of whom showed lower $S_{pO_{2}}$ among AMS^+^ individuals. Of note, our exploratory analyses revealed correlations between LLS symptoms at 4800 m and $S_{pO_{2}}$ values from the preceding days at lower altitudes, which is in agreement with work from Leichtfried et al. (2016), who showed similar prospective correlations. In addition, significant differences were observed between AMS^+^ versus AMS^–^ individuals for nocturnal $S_{pO_{2}}$ variance, ODI and TST < 80% $S_{pO_{2}}$. Nevertheless, despite these statistically significant effects, we also acknowledge the overlap between AMS^+^ and AMS^−^ groups for most of the overnight parameters, which limits the clinical relevance of these findings and highlights the need for more research to refine and improve the sensitivity of these measures for detecting AMS susceptibility.

The ODI has previously demonstrated strong agreement with the apnoea–hypopnoea index (Torre‐Bouscoulet et al., 2007), a quantitative marker for the occurrence and severity of sleep‐disordered breathing, which is known to occur with ascent to altitude (Ainslie et al., 2013). Previous studies have shown elevations in the apnoea–hypopnoea index in the presence of reduced arousals, and no changes in ODI (Nussbaumer‐Ochsner et al., 2012) nor significant improvement in periodic breathing (Bloch et al., 2010) with acclimatization. This is in contrast to the present findings, which show a reduction in ODI with partial acclimatization (day 11 vs. day 13 nights at top camp), and with this coinciding with reductions in AMS symptoms. The latter is in further contrast to the existing literature, which has demonstrated no differences between control and AMS (or high‐altitude pulmonary oedema) groups for ODI or the number of desaturations (Eichenberger et al., 1996; Erba et al., 2004). One explanation for a discrepancy between this study and the literature could be attributable to the introduction of acetazolamide in select individuals during this period; however, the degree to which such limited acetazolamide administration influenced this result is unclear. Nevertheless, the observed difference between AMS^−^ and AMS^+^ subjects for TST < 80% $S_{pO_{2}}$ was consistent with that observed by Erba et al. (2004) for TST < 70% $S_{pO_{2}}$. Likewise, the relationships between hypoxic burden and LLS were novel and consistent with findings from clinical studies related to sleep apnoea (Chen et al., 2018).

Last, the significance of the findings from the exploratory results was twofold: (1) various overnight oximetry metrics were correlated with the presentation of AMS symptoms the following morning, and oximetry from days prior was related to the LLS_peak_ that occurred days later; and (2) LLS did not exhibit these same determinant relationships (refer to Table 3). Taken together, this reinforces the evidence that overnight oximetry could be predictive of AMS susceptibility and severity days before peak symptoms present. The relative consistency of the correlations across the different portions of data analysis highlights that regardless of where you take the overnight data from, there might be a predictive value for AMS susceptibility. Moreover, these exploratory data indicate that there might be a portion of the end of sleep (immediately before waking) or early morning (before rising) that might be as useful or more useful for predicting and detecting AMS; however, further investigation is required to confirm this observation. Nevertheless, despite these overnight oximetry observations showing some potential to predict AMS susceptibility, we advise caution regarding the generalizability of our findings, which are limited to similar ascent profiles and conditions (i.e., low‐risk setting for AMS with ≤400 m increase above 2000 m).

### 4.2 Limitations and future directions

The inability to control for extraneous variables (e.g., activity, sleep and environmental conditions) and the low participant numbers were weaknesses of the present study but are common limitations for such field‐based research (Ainslie, 2014). We also acknowledge the fact that $S_{aO_{2}}$ measurements were not collected simultaneously with morning and overnight $S_{pO_{2}}$ measurements, which could have impacted agreement between measurements.

Likewise, the strength of the relationships observed between LLS and $S_{pO_{2}}$ metrics might have been influenced by the study being conducted at the ‘threshold’ of AMS symptoms, as previously described (i.e., not high enough LLS or altitude) (Ross et al., 2013). Cut‐off values described herein should be reviewed with caution because they are estimated across the expedition rather than at each altitude, not accounting for the rate of ascent (or potential for acclimatization). As such, future research with a larger number of participants and across several mountain ranges would be advantageous for validating the accuracy and precision of pulse oximetry as a diagnostic/predictive marker for AMS, with additional investigations into any need for the application of correction factors to cut‐off ranges. Likewise, we acknowledge that age could be a potential confounding factor; therefore, any future predictive studies should take this into account.

Future studies might also build upon the present findings to develop a predictive model that incorporates multiple nocturnal oximetry metrics chosen by calculated feature‐selection techniques (Alvarez et al., 2010), which could be used prospectively to predict those at risk for clinical deterioration in AMS. It is worth noting that such robust prediction models would be possible only with the use of overnight pulse oximetry, which enables the collection of a variety of additional metrics that have the potential to improve predictions (as outlined in this study). Also, such modelling might benefit from extracting and assessing additional conventional metrics [e.g., $S_{pO_{2}}$ delta index (Pépin et al., 1991) and coefficient of variation for $S_{pO_{2}}$ (Tellez et al., 2016)] and non‐linear metrics [e.g., sample entropy, approximate entropy (Richman & Moorman, 2000), Lempel Ziv (Lempel & Ziv, 1976) and central tendency measure (Alvarez et al., 2007)], which were not evaluated here. Spectral analysis or analyses within the frequency domain (e.g., frequency band analysis) for overnight oximetry might also prove useful when applied to data collected at altitude (Alvarez et al., 2010).

## 5 CONCLUSION

Findings from the present study demonstrate the potential of nocturnal oximetry for the detection and prediction of AMS or of those who are acclimatizing poorly during ascent to altitude. Furthermore, measurements of nocturnal $S_{pO_{2}}$ during ascent might help to avoid underestimations of hypoxaemia that are encountered overnight, and graphical representations of nocturnal data described herein might help to marry utility and practicality for nocturnal oximetry measurements during ascent. Further investigations are required to determine whether predictive modelling incorporating multiple metrics simultaneously could improve predictive capabilities of nocturnal pulse oximetry for AMS during ascent to altitude and would be highly beneficial for the safety and success of climbers/trekkers.

## AUTHOR CONTRIBUTIONS

Concept: Kelsey E. Joyce and Samuel J. E. Lucas. Design: Kelsey E. Joyce, John P. Delamere and Arthur R. Bradwell. Data collection: Christopher T. Lewis, Chris Bradley, Owen Thomas, Kelsey E. Joyce, Kimberly Ashdown, and Rebekah A. I. Lucas. Statistical analysis: Kelsey E. Joyce. Writing: Kelsey E. Joyce, Kimberly Ashdown, Christopher T. Lewis, Chris Bradley, Owen Thomas, and Rebekah A. I. Lucas. Clinical oversight: Abigail Letchford and Will Malein. All authors approved the final version of the manuscript and agree to be accountable for all aspects of the work in ensuring that questions related to the accuracy or integrity of any part of the work are appropriately investigated and resolved. All persons designated as authors qualify for authorship, and all those who qualify for authorship are listed.

## CONFLICT OF INTEREST

The authors declare no conflicts of interest.

## Supporting information




**FIGURE S1A** Relative (a) and cumulative (b) frequency distribution plots. Plots presented for nocturnal oxygenation ($S_{pO_{2}}$) data collected from a single individual are for select nights (for clarity). ‘Worsened’ skewness (first vs. second night at top camp: 1.28 vs. 0.20; indicated by green and purple lines) and kurtosis proper are demonstrated during two successive nights at 4800 m and were evident from the change in ‘peakedness’ and inflection of the peak. Plotted data were representative of an individual who scored positive for acute mountain sickness (via Lake Louise scores) twice during residence at 4800 m and later required premature descent. Taken together, these data illustrate, and uniquely depict, poor tolerance to altitude.
**FIGURE S1B** Relative (c) and cumulative (d) frequency distribution plots of nocturnal oxygenation ($\%S_{pO_{2}}$). Plots are presented for a single individual from select nights (for clarity), albeit for an individual who scored negative for acute mountain sickness (AMS) throughout the expedition. In contrast to the AMS^+^ individual from Figure S1A, improvements in skewness and kurtosis were observed on the second night at the top camp (purple vs. green lines) compared with the first night. Thus, these data illustrate, and uniquely depict, altitude tolerance.

## Data Availability

Data that support these findings are available upon reasonable request from the corresponding author. Data are not publicly available for privacy reasons.
